# Long-lasting neutralizing antibodies and T cell response after the third dose of mRNA anti-SARS-CoV-2 vaccine in multiple sclerosis

**DOI:** 10.3389/fimmu.2023.1205879

**Published:** 2023-06-19

**Authors:** Alessandro Maglione, Rachele Francese, Irene Arduino, Rachele Rosso, Manuela Matta, Simona Rolla, David Lembo, Marinella Clerico

**Affiliations:** ^1^ Laboratory of Neuroimmunology, Department of Clinical and Biological Sciences, University of Turin, Orbassano, Italy; ^2^ Laboratory of Molecular Virology and Antiviral Research, Department of Clinical and Biological Sciences, University of Turin, Orbassano, Italy; ^3^ San Luigi Gonzaga University Hospital, Orbassano, Italy

**Keywords:** anti-SARS-CoV-2 vaccination, multiple sclerosis, COVID-19, neutralizing antibodies, T-cell response, disease modifying therapies

## Abstract

**Background and objectives:**

Long lasting immune response to anti-SARS-CoV-2 vaccination in people with Multiple Sclerosis (pwMS) is still largely unexplored. Our study aimed at evaluating the persistence of the elicited amount of neutralizing antibodies (Ab), their activity and T cell response after three doses of anti-SARS-CoV-2 vaccine in pwMS.

**Methods:**

We performed a prospective observational study in pwMS undergoing SARS-CoV-2 mRNA vaccinations. Anti-Region Binding Domain (anti-RBD) of the spike (S) protein immunoglobulin G (IgG) titers were measured by ELISA. The neutralization efficacy of collected sera was measured by SARS-CoV-2 pseudovirion-based neutralization assay. The frequency of Spike-specific IFNγ-producing CD4+ and CD8+ T cells was measured by stimulating Peripheral Blood Mononuclear Cells (PBMCs) with a pool of peptides covering the complete protein coding sequence of the SARS-CoV-2 S.

**Results:**

Blood samples from 70 pwMS (11 untreated pwMS, 11 under dimethyl fumarate, 9 under interferon-γ, 6 under alemtuzumab, 8 under cladribine, 12 under fingolimod and 13 under ocrelizumab) and 24 healthy donors were collected before and up to six months after three vaccine doses. Overall, anti-SARS-CoV-2 mRNA vaccine elicited comparable levels of anti-RBD IgGs, neutralizing activity and anti-S T cell response both in untreated, treated pwMS and HD that last six months after vaccination. An exception was represented by ocrelizumab-treated pwMS that showed reduced levels of IgGs (p<0.0001) and a neutralizing activity under the limit of detection (p<0.001) compared to untreated pwMS. Considering the occurrence of a SARS-CoV-2 infection after vaccination, the Ab neutralizing efficacy (p=0.04), as well as CD4+ (p=0.016) and CD8+ (p=0.04) S-specific T cells, increased in treated COVID+ pwMS compared to uninfected treated pwMS at 6 months after vaccination.

**Discussion:**

Our follow-up provides a detailed evaluation of Ab, especially in terms of neutralizing activity, and T cell responses after anti-SARS-CoV-2 vaccination in MS context, over time, considering a wide number of therapies, and eventually breakthrough infection. Altogether, our observations highlight the vaccine response data to current protocols in pwMS and underline the necessity to carefully follow-up anti-CD20- treated patients for higher risk of breakthrough infections. Our study may provide useful information to refine future vaccination strategies in pwMS.

## Introduction

The mRNA vaccines rapidly became the most used to counteract severe acute respiratory syndrome coronavirus 2 (SARS-CoV-2) spread (1) especially in frail subjects such as people with Multiple Sclerosis (pwMS). Whereas vaccination showed an adequate safety profile (2) and high efficacy in preventing COVID-19 transmission and severe disease outcomes in immunocompetent people (3, 4), pwMS are usually treated to prevent or block inflammation with disease-modifying therapies (DMTs) that modulate the immune system and, consequently, may lead to a suboptimal response to vaccination and increased probability of infection/re-infection (5–7). Several studies have shown that high-efficacy DMTs induced a weak immune response to anti-SARS-CoV-2 vaccination in pwMS: after two doses of mRNA vaccines, pwMS treated with ocrelizumab (anti-CD20 therapy) and fingolimod (sphingosine-1-phosphate receptor modulator) showed reduced levels of anti-SARS-CoV-2 spike IgG compared to healthy individuals and pwMS under other treatments (8–12). Due to humoral response decrease over 6 months following the second vaccine dose, authorities suggested the booster dose (10). Despite an increase in seroconversion after the booster (or third) dose, anti-SARS-CoV-2 spike IgG levels are still reduced in pwMS under anti-CD20 or sphingosine-1-phosphate receptor modulators (2, 13–15).

Antibodies (Ab) directed toward the Receptor Binding Domain (RBD) of the SARS-CoV-2 Spike (S) protein are widely considered to be a good representation of the Ab neutralizing activity as they positively correlate with SARS-CoV-2 neutralizing Ab measured in neutralization assays (16, 17). ELISA-based tests present advantages such as low cost, speed, and safety, but only Ab that block the RBD/ACE2 interaction are detected, thus both the actual neutralizing activity and the presence of non-RBD binding Ab, which may also be neutralizing, are missing (18–21). The most direct methods to evaluate the neutralizing Ab induced by SARS-CoV-2 vaccination and predict their function and efficacy are the live virus-based or alternatively the pseudovirion-based infection inhibition tests. As opposed to the use of live virus, neutralization tests with pseudovirions can be easily carried out in BSL-2 conditions and the presence of a reporter gene enables an objective, rapid and quantitative detection (21, 22). To the best of our knowledge, only one report investigated the Ab neutralizing activity with the above-mentioned methods in the context of pwMS (23). Therefore, the actual Ab neutralizing response in pwMS still remains an open question.

The longevity of elicited immunity after the third dose of anti-SARS-CoV-2 vaccination is currently under investigation. A study on healthcare workers showed that reduction in Ab levels 5 months after the third vaccine dose was slower than after the second (24), while a mid/long-term follow-up of the immune response after booster vaccination dose in pwMS is missing to date. Moreover, data indicate that immunity induced by anti-SARS-CoV-2 vaccination is mediated both by neutralizing Ab that block infection by preventing viral entry into host cells, and cellular immunity that rapidly activates once the infection has occurred, hence protecting from severe disease (25). Actually, low neutralizing Ab levels are a relevant risk factor for breakthrough infection risk in pwMS (6) while SARS-CoV-2 antigen-specific T cell response seems to be preserved in the majority of pwMS (15, 26–28).

Here, 70 pwMS and 24 healthy donors (HD) were followed up for 6 months after three vaccination doses to evaluate long-term Ab neutralizing activity and T cells response. Humoral response was evaluated by both anti-RBD IgG titration and neutralization assay using SARS-CoV-2 pseudovirions. Antigen-specific T cell response was quantified by *in vitro* restimulation of Peripheral Blood Mononuclear Cells (PBMCs) with S peptides. Our findings provide additional information to refine future vaccination strategies in MS patients.

## Materials and methods

### Subjects

PwMS and HD, belonging to this prospective single-center study, were recruited at the AOU San Luigi Gonzaga, Orbassano (TO, Italy) according to the following inclusion/exclusion criteria. A diagnosis of MS, according to the most recently revised McDonald criteria (29), and eligibility for anti-SARS-CoV-2 vaccination were considered as inclusion criteria. Any medical condition that does not allow the signing of informed consent and a prior history of symptomatic SARS-CoV-2 infection or breakthrough infection before the third dose were considered as exclusion criteria. All the subjects in the study were vaccinated with two doses of Comirnaty (ex mRNA BNT162b2) mRNA vaccine (Pfizer/BioNTech Inc, BioNTech Manufacturing GmbH) and then with the third dose (booster) of Comirnaty or Spikevax (ex mRNA-1273) vaccine (Moderna, Moderna Biotech Spain S.L.). COVID-19 disease was not reported from any of the subjects before vaccination. COVID-19 infection after vaccination was determined by self-reported positive COVID-19 test during the follow-up and/or presence of nucleocapsid Ab (Anti-N) in collected serum samples.

### Blood and sera collection

Blood and sera were collected immediately before the first dose of Comirnaty vaccine (Pfizer/BioNTech Inc, BioNTech Manufacturing GmbH) (P0), 4 weeks ( ± 15 days) (P1) and 6 months ( ± 15 days) (P6) after the booster vaccination. Sera were immediately frozen for further analysis. PBMCs were isolated by density gradient centrifugation using Histopaque-1077 (Sigma-Aldrich, St. Louis, MO, USA) from heparinized venous blood.

### Anti-SARS-Cov2 ELISA

Anti-RBD IgG titers were measured with the SARS-CoV-2 RBD IgG ELISA (EIA-6150, DRG Diagnostics, Marburg, Germany, EU; lot number 142K061) following manufacturer instructions. Results are expressed in IU/ml (log10), and the cut-off threshold corresponded to 1.4 IU/ml (log10), according to manufacturer indications. Anti-N IgG were measured with the SARS-CoV-2 (COVID-19) IgG ELISA test (NOVATEC Immunodiagnostica GmBH, Dietzenbach, Germany, EU; lot number COVG-053) according to manufacturer method.

### Cell lines

The human embryonic kidney cells (293T, ATCC, CRL-3216), the baby hamster kidney cells (BHK21, ATCC, C-13) and the hepatocyte derived cellular carcinoma cell line (Huh7) (ECACC, Cat num: 01042712) were grown as monolayers in Dulbecco’s modified Eagle’s medium (DMEM) (Sigma-Aldrich St. Louis, MO, USA), supplemented with 10% (v/v) heat inactivated fetal bovine serum (FBS) (Sigma-Aldrich) and a 1% (v/v) antibiotic solution (penicillin–streptomycin, Sigma-Aldrich) at 37˚C in 5% CO2 atmosphere.

### SARS-CoV-2 pseudovirion production, titration and characterization

SARS-CoV-2 pseudotyped viruses were produced and titrated according to Nie et al. (21) and were analyzed by means of Western Blot analysis to verify the presence of the SARS-CoV-2 S protein on the VSV envelope (30). The detailed protocols are reported in the **Supplementary Materials**.

### SARS-Cov-2 pseudovirion-based neutralization assay

The neutralization assay was performed according to Almahboub et al. (31) and Nie et al. (21). Huh7 cells were pre-seeded in a 96 well/plate at a density of 1.2x10^4^ cell/well. The following day, serum samples were heat inactivated at 56°C for 30 min in a water bath. Then, a fixed amount of SARS-CoV-2 pseudovirions (650 TCID_50_/well) (21) was incubated with serial dilutions of serum samples (from 1:20 to 1:14580 in duplicate) for 1h at 37°C in continuous oscillation. As controls, six wells were incubated with only culture medium (CC wells) and six wells were infected but not treated (VC wells). After 1h incubation, the pre-treated virus was inoculated on Huh7 cells for 24h at 37°C to evaluate the residual viral infectivity. The detection was performed by adding the Britelite plus reporter gene assay system (PerkinElmer) to cells in a 1:1 ratio with the culture medium, for 2 min in the darkness at RT. 150µl of each well were then transferred to a corresponding 96-well chemiluminescence detection plate and the RLU were read in the Infinite F200 luminescence reader (TECAN). Inhibition (%) of luciferase activity from each serum dilution was calculated as follows: *100 - [(mean RLU of each sample - mean RLU of CC)/(mean RLU of VC - mean RLU of CC) x 100]*. Inhibition (%) were then plotted against each dilution using four-parameter logistic (4PL) curve, and 50% inhibitory dilution (ID_50_) values for each sample were calculated using GraphPad Prism software, version 8.0 (GraphPad Software, San Diego, CA, USA). As recommended by the World Health Organization (WHO) (29), the neutralization assay was calibrated and validated with the Working Standard Reagent for anti-SARS-CoV-2 immunoglobulin (National Institute for Biological Standards and Controls –NIBSC-, code: 21/234), that was also employed as positive control at each run of the experiment. As negative control, a serum sample from an uninfected and unvaccinated person was used.

### Evaluation of T cells response

PBMCs were cultured at 10^7^/mL in RPMI-1640 Medium (Sigma-Aldrich, St. Louis, MO, USA) supplemented with 10% FBS (Corning, New York, NY, USA) and stimulated or not with PepTivator^®^ SARS-CoV-2 Prot_S Complete (Miltenyi Biotec, Bergisch Gladbach, Germany, EU) at a final concentration of 0.6 nmol of each peptide/mL. PepTivator SARS-CoV-2 Prot_S Complete is a pool of lyophilized peptides, covering the complete protein coding sequence (aa 5–1273) of the surface or S glycoprotein of SARS-CoV-2 (GenBank MN908947.3, Protein QHD43416.1). Cells were incubated at 37°C for two hours and then 5μg/mL of Brefeldin A (Sigma-Aldrich, St. Louis, MO, USA) was added to cells to allow intracellular cytokine staining. PBMCs were incubated for further 16 hours and then prepared for staining. To detect anti-S specific CD4 and CD8 T cells, stimulated cells were stained for the surface antigen CD4 and CD8 (BioLegend, San Diego, CA, USA); fixed with Cyto-Fast Fix/Perm Buffer Set (BioLegend) and intracellular stained with anti-IFN-γ mAb (BioLegend). Stained PBMCs were acquired on CELESTA FACS (BD Biosciences, San Jose, CA, USA) and analyzed with FlowJo software (Ashland, OR, USA) Version 10. 50.000 CD4+ events were acquired and analyzed. The frequency of Spike-specific IFNγ-CD4+ and CD8+ T cells was obtained by subtracting cytokine background obtained from unstimulated cells.

### Standard protocol approvals, registrations, and patient consents

This study obtained ethics approval from the ethics committee of AOU San Luigi Gonzaga, Orbassano (TO), Italy; Ref. number #117-2021). All the subjects included in the study consented to participate in the study.

### Data availability

Data sets used during this study are available from the corresponding author on reasonable request.

### Statistics

Anti-RBD IgGs titers and ID_50_ values of the inhibition curves were calculated by a regression analysis using GraphPad Prism software, version 8.0 (GraphPad Software, San Diego, CA, USA) by fitting a quadratic curve and a variable slope-sigmoidal dose-response curve and statistically compared with the F-test, respectively. Ab levels were transformed on a log10 scale, to normalize their distribution and according to previous literature (12, 14).

Statistical analysis was performed using ANOVA Analysis of variance followed by Bonferroni post-test or t-test as reported in the legends to the Figures. Multivariable analysis was performed using a linear regression model computed by R version 3.6.3 (2020-02-29). Model was used to compare log-transformed P1 Anti-RBD IgG titer across patients treated with different DMTs, after adjusting for age, sex, EDSS score, MS type: relapsing remitting MS (RRMS), secondary progressive MS (SPMS), primary progressive MS (PPMS), MS disease duration, booster type (Comirnaty/Spikevax/COVID-19), Ab levels in the P0 samples. P1 Ab titers were included in the model to compare log-transformed P6 Anti-RBD IgG titers across subjects.

## Results

### Anti-RBD IgGs titers persist up to six months after SARS-CoV-2 vaccination in pwMS

11 untreated pwMS, 59 pwMS under different DMTs and 24 HD were recruited and prospectively followed-up from their first shot of anti-COVID-19 mRNA vaccine (Pfizer) to 6 months after the third dose (Pfizer/Moderna). Demographic and clinical characteristics are reported in **Table 1**.

**Table 1:** Clinical characteristics.

Therapy	HD (n=24)	NT MS (n=11)	T MS (n= 59)					
			DMF (n=11)	IFN (n=9)	ALEM (n=6)	CLAD (n=8)	FING (n=12)	OCR (n=13)
**Sex (F/M)**	20/6	11/0	5/6	4/5	4/2	5/2	10/2	10/3
**Age**	43.5 (32.3 – 50.3)	51 (47.5 – 63.5)	39 (38 – 48.5)	47 (43 – 55)	37 (36.3 – 47.5)	46.5 (42.8 – 51.8)	52 (46.3 – 59.3)	57 (53 – 64)
**MS disease duration (years)**	NA	8 (3 –17.5)	6 (5 – 0)	14 (10 – 19)	13.5 (9.5 – 2)	10 (1.8 – 13.5)	18.5 (7 – 24)	12 (9 – 22)
**EDSS**	NA	2 (1 – 2)	1 (0.5 – 1.25)	1 (1 – 1.5)	3 (1.6 – 5.5)	2.8 (2 – 3.9)	4 (2 – 6.5)	5.5 (3.5 – 6.5)
MS type								
RRMS	–	8	11	9	4	7	7	9
SPMS	–	1	–	–	2	1	5	3
PPMS	–	2	–	–	–	–	–	1
Booster type								
Spikevax	8	5	3	8	–	3	4	4
Comirnaty	16	6	8	1	6	5	8	9
COVID-19 infection between P1 and P6								
COVID-19 -	15	7	10	9	3	4	8	9
COVID-19 +	9	4	1	–	3	4	4	4
Relapses								
No relapses	NA	10	11	–	3	7	11	12
After two doses	NA	1	–	–	2	1	–	1
After three doses	NA	–	–	–	1	–	1	–
**Time between last infusion of depletive agents and vaccination (months)**	NA	NA	NA	NA	45 (40-45)	6.4 (6-10)	NA	4.4 (3-7.6)

Results are expressed as Median and Inter-quartile range (IQR). HD, healthy donors; NT, not treated; DMF, dimethyl fumarate; IFN, Interferon; ALEM, Alemtuzumab; CLAD, Cladribine; FING, Fingolimod; OCR, Ocrelizumab.NA = not applicable.

The titer of anti-RBD IgGs induced by the full cycle of anti-SARS-CoV-2 vaccination (three doses) was evaluated in serum samples collected immediately before vaccination (P0), one (P1) and six months (P6) after booster. Moreover, the anti-N Ab titration was performed to evaluate a response to the natural infection occurred after vaccination.

**Figure 1:**
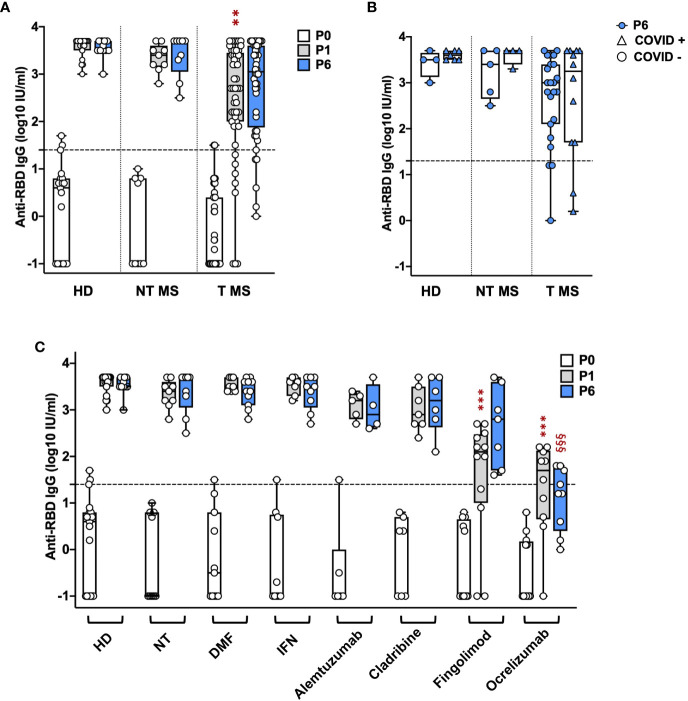
Kinetics of anti-RBD IgG levels in pwMS and HD. IgG titers have been compared between HD and untreated and treated pwMS **(A)**; then stratified by the occurrence of a natural infection after three doses of vaccine **(B)** or by specific therapy **(C)**. Anti-RBD IgGs have been quantified at three time points: immediately before vaccination (P0), one (P1) and six months (P6) after the third dose of vaccine. Dotted line corresponds to the cut-off threshold of 1.4 IU/ml (log10). **(A)** Statistic was performed by one-way ANOVA with Bonferroni correction for multiple comparisons. Asterisks correspond to p-value thresholds of one-way ANOVA with Bonferroni correction for multiple comparisons: * indicates HD at P1 vs. each other group at P1 (**p<0,002) **(B)** Subjects under interferon have been excluded from the analysis because no subjects under interferon experienced natural infection after vaccination. Statistic was assessed by the two-tailed unpaired t-test. **(C)** Statistic was performed by one-way ANOVA with Bonferroni correction for multiple comparisons. Different symbols have been used for different comparisons within each group: * indicates NT at P1 vs. each other group at P1 (***p<0.001); § indicates NT at P6 vs. each other group at P6(§§§p<0.001). HD, healthy donors; NT, not treated; T, treated, DMF, dimethyl fumarate; IFN, Interferon; COVID +, anti-N positive subjects; COVID -, anti-N negative subjects.

Treated (T) pwMS (2.4 ± 1.2; p = 0.001) showed a significant lower level of anti-RBD IgGs compared to HD (3.6 ± 0.2) at P1 while not treated (NT) pwMS (3.3 ± 0.3) showed comparable levels with HD (**Figure 1A**). At P6, no significant difference was observed comparing anti-RBD IgG levels in HD (3.5 ± 0.2) with NT pwMS (3.4 ± 0.5) and T pwMS (2.7 ± 1) (**Figure 1A**). Subsequently, pwMS were divided according to anti-N positivity and their anti-RBD IgGs level were compared (**Figure 1B**). No statistical difference was observed, suggesting that anti-RBD IgGs is not related to a possible natural infection after vaccination. PwMS under interferon were excluded from this analysis because none of these subjects experienced natural COVID-19 infection after vaccination.

To investigate the effect of therapies on anti-RBD IgGs, pwMS were then divided according to DMTs (**Figure 1C**). All T pwMS showed comparable levels of anti-RBD IgGs with exception of T pwMS under ocrelizumab (1.3 ± 1; p<0.0001) and fingolimod (1.6 ± 1.3; p=0.0009) that showed significant lower levels of Ab respect to NT pwMS (3.3 ± 0.3). This difference is maintained at P6 for pwMS under ocrelizumab (1.1 ± 0.7, p=<0.0001) compared to NT pwMS at P6 (3.4 ± 0.45). Interestingly, a significant difference in anti-RBD IgG titers was not observed between P1 and P6 within each group suggesting a long-lasting durability of anti-RBD IgGs.

Finally, the association of factors included in **Table 1** to anti-RBD IgG levels at P1 and P6 was explored by a multivariable regression analysis. Results of this analysis are reported in **Table 2**. Ab titers at P6 were significantly associated with P1 Ab level (p = 0.0099) and ocrelizumab therapy (p = 0.0012). We confirmed that anti-RBD IgG titers at P1 were associated with treatment with ocrelizumab (p < 0.00005) and fingolimod (p = 0.0005) which both showed significantly reduced anti-RBD Ab levels compared to NT pwMS. Moreover, anti-RBD IgG titers at P1 were significantly increased in subjects that had Spikevax booster with respect to Comirnaty (p = 0.0294). No association with any other considered factor was found.

**Table 2:** Multivariable analysis assessing factors associated with anti-RBD levels at P1 and P6.

Multivariable analysis P1					Multivariable analysis P6				
Variable	Beta coef.	Robust SE	p		Variable	Beta coef.	Robust SE	p	
					**Anti-RBD IgGs at P0**	2.67E-03	0.01725	0.8779	
**Anti-RBD IgGs at P0**	-1.73E-04	6.55E-04	0.7928		**Anti-RBD IgGs at P1**	5.19E-04	1.89E-04	0.0099	***
**Sex (Male vs. Female)**	-0.03953	0.666	0.9529		**Sex (Male vs. Female)**	-0.6582	0.6412	0.3128	
**Age (Years)**	8.11E-03	0.02606	0.7571		**Age (Years)**	-0.04094	0.02651	0.133	
**EDSS score**	9.13E-03	0.1716	0.9578		**EDSS score**	0.2179	0.1619	0.1885	
**MS disease duration (years)**	0.01051	0.03697	0.7775		**MS disease duration (years)**	-4.62E-03	0.03245	0.8878	
MS type					MS type				
RRMS	Ref.				RRMS	Ref.			
PPMS	1.297	1.036	0.2177		PPMS	0.5475	0.9328	0.5616	
SPMS	-0.6854	0.8333	0.4154		SPMS	-1.163	0.9888	0.2486	
Booster type					Booster type				
Comirnaty	Ref.				Comirnaty	Ref.			
Spikevax	1.161	0.5154	0.0294	*	Spikevax	0.3137	0.4456	0.4869	
Therapy					Therapy				
Not treated	Ref.				Not treated	Ref.			
Dimethyl fumarate	0.7545	0.6146	0.2262		Dimethyl fumarate	-0.1137	0.537	0.8337	
Interferon	0.03858	0.6325	0.9516		Interferon	-0.2345	0.6195	0.7078	
Alemtuzumab	0.3403	0.8722	0.6984		Alemtuzumab	-0.6895	0.9647	0.4803	
Cladribine	-0.5561	0.6361	0.3869		Cladribine	0.0202	0.9358	0.9829	
Fingolimod	-3.637	0.9721	0.0005	***	Fingolimod	-0.09006	1.409	0.9494	
Ocrelizumab	-4.446	0.936	<0.00005	***	Ocrelizumab	-3.544	0.9935	0.0012	**

p-values indicate a statistically significant relationship with the response variable in the model. Asterisks correspond to significance thresholds (***p<0.001; **p <0.02; *p<0.03).”Not treated” was chosen as the reference class (Ref.) for therapy, “Pfizer’’ was chosen as reference class for booster type and “RRMS” was chosen as reference class for MS type. EDSS, Expanded Disability Status Scale; RRMS, Relapsing Remitting Multiple Sclerosis; PPMS, Primary Progressive Multiple Sclerosis; SPMS, Secondary progressive Multiple Sclerosis.

### An efficient neutralizing response is present in the majority of pwMS over-time and is increased by natural infection in treated patients

**Figure 2:**
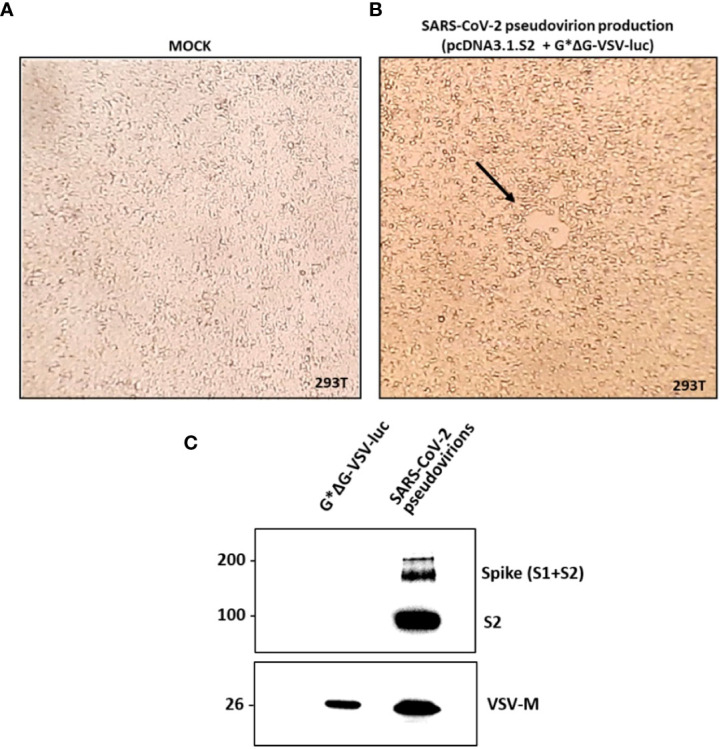
SARS-CoV-2 pseudovirion production and characterization. In **(A, B)** the SARS-CoV-2 pseudovirion production is reported. **(A)** Uninfected and untreated 293T cells (mock control). **(B)** 293T cells transfected with pcDNA3.1.S2 and infected with G*ΔG-VSV-luc observed under inverted microscope at 48h. The arrow highlights the observed syncytia. In **(C)** SARS-CoV-2 pseudovirion characterization by means of WB analysis is reported, showing the incorporation of the SARS-CoV-2 spike on the VSV virions.

SARS-CoV-2 pseudovirions were produced according to a previously reported protocol published on Nature Protocols by Nie et al. (21), which is briefly described in the Material and methods section. A viral stock with a titer corresponding to 1.5x10^5^ TCID_50_/ml was produced and used throughout the study. As reported by **Figures 2A, B**, a moderate to extensive cytopathic effect was observed in flasks transfected with pcDNA3.1.S2 plasmid and infected with the VSV-G pseudotyped virus after 48h or 72h, respectively. In order to verify the incorporation of SARS-CoV-2 spike glycoprotein on the VSV particles, pseudovirion production was characterized by means of western blot analysis. As reported in **Figure 2C**, the S protein was efficiently expressed on pseudovirion envelop: specific bands were detected in the lane of SARS-CoV-2 pseudovirions, whilst no specific band was found in the VSV-G pseudovirions (generated with the same procedure as SARS-CoV-2 pseudovirions) in the corresponding position. The monomer of the S protein (S1 + S2) was observed at a position of about 190 kDa and the S2 domain was detected at 90kDa. The SARS-CoV-2 pseudovirions, together with G*ΔG-VSV, were tested against a VSV-M specific Ab, showing a common band at 26kDa. Altogether, our results indicated that we generated a well-defined SARS-CoV-2 pseudovirion production suitable for the neutralization assays.

**Figure 3:**
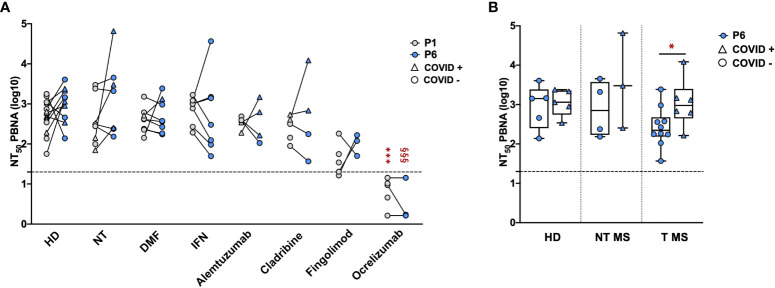
Neutralizing activity of serum samples from pwMS and HD. Neutralization titer has been compared between HD and pwMS stratified by therapy **(A)** or by the occurrence of a natural infection after three doses of vaccine **(B)**. Neutralization assay were performed at two time points: one (P1) and six months (P6) after the third vaccination dose. Dotted line corresponds to the cut-off threshold of 1.3 NT_50_ (log10). **(A)** Statistical significance was assessed by one-way ANOVA with Bonferroni correction for multiple comparisons. Different symbols were used for different comparisons within each group: * indicates NT at P1 vs. each other group at P1 (***p<0,001); § indicates NT at P6 vs. each other group at P6 (§§§ p<0.001). **(B)** Statistical significance was assessed by two-tailed unpaired t-test within each group (* p<0.03). Subjects under Interferon were excluded from the analysis because no subjects under interferon experienced natural infection after vaccination. All the results are presented as the mean values of two independent experiments. NT_50_ PBNA, neutralizing titer 50 calculated with pseudovirion based neutralization assay; HD, healthy donors; NT, not treated; T, treated; DMF, dimethyl fumarate; IFN, Interferon; COVID +, anti-N positive subjects; COVID -, anti-N negative subjects.

The serum samples collected at P1 and P6 were subsequently tested by means of the neutralization assay, in order to directly evaluate the Ab function and efficacy against SARS-CoV-2 pseudovirions. Focusing on sera collected at P1, we compared the neutralizing activity of samples from pwMS with that of HD (**Figure 3A**, grey dots). As reported, no statistical difference in the neutralizing activity was observed between HD (2.7 ± 0.4) and NT pwMS (2.5 ± 0.6) or pwMS under different therapies, with exception of pwMS under ocrelizumab. As expected, considering the mechanism of action of ocrelizumab and the previously reported low levels of anti-RBD Ab, a significant reduction of the neutralizing activity was observed in pwMS under anti-CD20 therapy (0.8 ± 0.4; p=0.0001) compared to NT pwMS (2.5 ± 0.6). In particular, despite a small production of anti-RBD Ab, the neutralizing activity of sera from ocrelizumab-treated MS patients was always under the limit of detection (i.e NT_50_ <1.3), indicating an absence of neutralization capacity.

Similar results were observed focusing on sera collected at P6 (**Figure 3A**, blue dots). No statistical difference in the neutralizing activity at P6 was observed between HD (3.0 ± 0.4) and untreated pwMS (3.2 ± 0.9) or pwMS under different therapies, with the exception of pwMS treated with ocrelizumab (0.5 ± 0.5; p< 0.001) showing a neutralizing activity always under the limit of detection. Additionally, the potential reduction of the neutralizing ability after several months from the three doses was evaluated. As reported in **Figure 3A**, we didn’t observe significant differences comparing the neutralizing titers at P1 and P6 within each group, suggesting that, where present, the neutralization activity against SARS-CoV-2 is maintained over time. A difference in neutralizing efficacy is visible at P6 comparing T pwMS in which a natural SARS-CoV-2 infection occurred after vaccination (3 ± 0.6, p = 0.04) with uninfected T pwMS (2.4 ± 0.5) (**Figure 3B**), suggesting that natural infection may increase neutralizing response in these subjects. However, the same difference is not visible in HD and NT pwMS. Similarly to what was previously done for anti-RBD quantification, we excluded pwMS under interferon from this analysis because none of these subjects got COVID-19 after vaccination.

**Figure 4:**
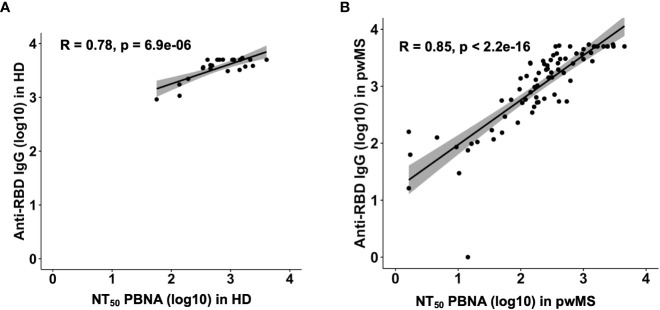
Correlation plots between anti-RDB IgG levels and neutralizing titer 50 in HD **(A)** and pwMS **(B)**. Plots have been generated using values from all groups. Pearson correlation coefficients were computed to assess the linear relationship between Anti-RDB IgG levels and NT_50_.

Overall, we observed a robust correlation between the previously reported anti-RBD Ab levels and the Ab neutralizing efficacy in HD (Pearson correlation; R=0.78, p= 6.9e-06) and in pwMS (Pearson correlation; R=0.85, p< 2.2e-16) (**Figure 4**).

### PwMS display a good spike-specific CD4+ and CD8+ T immune response that is increased by COVID-19 and is independent of DMTs

**Figure 5:**
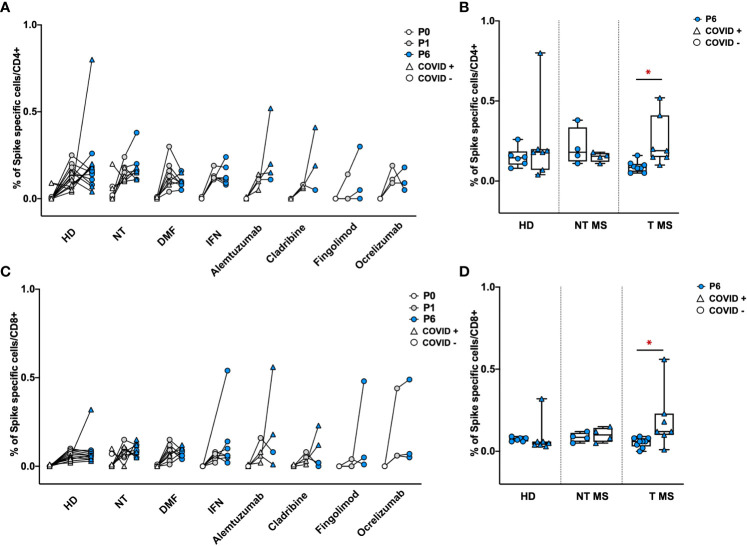
Frequency of Spike specific CD4+ and CD8+ T cells in PBMCs of pwMS and HD. Percentage of Spike specific CD4+ and CD8+ T cells has been obtained by *in-vitro* restimulation of PBMCs with Spike peptides, followed by intracellular staining for IFNγ. The percentage of Spike specific CD4+ **(A, B)** or CD8+ T **(C, D)** cells was obtained by subtracting values of unstimulated cells. Obtained percentage were compared between HD and pwMS stratified by therapy **(A, C)** or by the occurrence of a natural infection after three doses of vaccine **(B, D)**. Statistics were assessed by two-tailed unpaired t-test (*p<0.03). HD, healthy donors; NT, not treated; T, treated; DMF, dimethyl fumarate; IFN, Interferon; COVID +, anti-N positive subjects; COVID -, anti-N negative subjects.

To determine the levels of S-specific T-cell activity, the number of CD4+ and CD8+ cells releasing IFNγ was assessed by cytofluorimetry after exposure of PBMCs to a 15-mer peptide pool covering the S protein of Wuhan wild-type SARS-CoV-2 (**Supplementary Figure 1**). One and six months after vaccination, all groups of pwMS showed a similar frequency of S-specific IFNγ producing- CD4+ and CD8+ T cells comparable to that of HD (**Figures 5A, C**). Notably, T pwMS in which occurred a natural COVID-19 infection after vaccination display a higher frequency of both CD4 (0.24% ± 0.15; p=0.016) and CD8+(0.19% ± 0.18; p= 0.04) S-specific T cells response compared to T pwMS who remains protected from the infection (0.09% ± 0.03 and 0.05% ± 0.02 respectively, **Figures 5B, D**). These results suggest that COVID-19 disease may increase the S-specific T cells repertoire, more than the vaccination alone.

## Discussion

Here we report the results of our observational, monocentric, prospective cohort study on SARS-CoV-2 vaccinated pwMS and HD followed up to 6 months after the third dose, in terms of elicited humoral and T cell responses, with a special focus on the neutralizing activity of Abs. This cohort is extremely peculiar as only subjects receiving the three doses of anti-SARS-CoV-2 vaccine without prior or breakthrough SARS-CoV-2 infection were included, allowing us to characterize the specific immune response to a well-defined new antigen in pwMS and to address several unmet clinical questions on the immune response to eventual natural infections in pwMS under DMTs.

Regarding humoral immunity, our main result indicated that after three doses of anti-SARS-CoV-2 mRNA vaccine, pwMS develop a significant Ab response. This result is concordant with previous observations (8, 32–34), albeit obtained with a different methodology of Ab quantification as CLIA (32–34), or for a different target as recombinant S1 subunit (8) or trimeric S (34) instead of RBD region. In line with other studies (2, 13–15), we observed a weak anti-RBD IgG production still after the third dose in pwMS under ocrelizumab and fingolimod, even if the booster dose was able to induce seroconversion in several patients (2, 13–15).

A key observation was the maintenance after six-months of high anti-RBD IgG levels after three doses not only in our cohort of HD, similarly to what was observed in a study on healthcare workers (24), but also in the majority of pwMS. The increasing trend between P1 and P6 in the fingolimod group could be due to intercurrent SARS-CoV-2 infection as the mean growth of Ab titers between P1 and P6 was ten folder higher in pwMS COVID-19+ compared to COVID-19-. PwMS under ocrelizumab showed the lowest levels of Ab at 6 months after the third dose compared to differently treated pwMS, suggesting that these subjects are more at risk of a breakthrough COVID-19 infection (6). Indeed, low neutralizing Ab levels are a relevant risk factor for breakthrough infections in pwMS, since neutralizing Ab prevent viral entry into the host cell (6). On the other hand, cellular immunity protects from severe disease (25). Our results on cellular immunity showed comparable levels of spike-specific IFNγ-producing CD4+ and CD8+ T cell among pwMS, confirming that antigen-specific T cell response seems to be preserved in pwMS under anti-CD20 treatment, as reported by previous studies (26, 27). We did not observe a reduced S-specific T cell response in pwMS under fingolimod, as reported by other studies (15, 28, 35), but this is not surprising as other studies on influenza vaccination showed that pwMS under fingolimod are able to elicit a T cell response similar to HD (36).

With the aim of investigating deeply the humoral response, we evaluated not only the anti-RBD IgG levels, which are considered a good representation of the Ab neutralizing activity (16, 17), but also the neutralizing Ab (nAb) function and efficacy by means of a pseudovirion-based neutralization assay. The use in a MS context of a novel, sensitive and high-throughput pseudovirion-based assay, which allows the direct evaluation of the nAb function and that directly correlates with a live virus neutralization assay, is one of the strengths of our work (21, 37). So far, a small number of studies have investigated the Ab neutralizing activity through this method (23), whereas the majority of studies employed the analysis of the anti-RBD IgGs levels as surrogate of the direct evaluation of the Ab neutralizing activity (8, 9, 12). Herein, we found a good correlation between the neutralizing activity and the anti-RBD levels both in HD (as expected) (17) and in pwMS group, showing R-values of 0.78 and 0.85 respectively. The results obtained with the evaluation of the anti-RBD IgG levels were confirmed with the pseudovirion-based assay: no statistical difference in the neutralizing activity was observed between HD and pwMS under all the considered therapies, with the exception of ocrelizumab, and the protective capacity was maintained over time (six-month observation). Nevertheless, the direct analysis of Ab neutralization allowed us to highlight novel aspects of the vaccination response in a MS context. Differently from what we observed from the analysis of the anti-RBD levels, we did not observe a statistically reduced neutralizing response in fingolimod-treated patients, thus indicating that despite a reduced number of Ab, a partial ability in neutralizing the virus is maintained. Consistently with our findings, Gyang et al., through a neutralization assay based on SARS-CoV-2 pseudotyped lentivirus, demonstrated that pwMS under B-cell depleting therapies (rituximab and ocrelizumab) have a reduced neutralizing response compared to other pwMS, which correlated with the time from the last anti-CD20 infusion. Additionally, the authors showed that prior COVID-19 illness, DMT category, and pyramidal function were significant predictors of vaccine responsiveness, and that circulating absolute lymphocyte count (ALC) and IgG levels correlated with neutralizing Ab levels (23).

We additionally investigated how the occurrence of SARS-CoV-2 natural infection after the third vaccination dose affected the immune response in pwMS and HD. We found no differences in anti-RBD IgG amount between P1 and P6 suggesting that anti-RBD IgG might not be significantly increased by a natural infection. Despite this result, natural infection acquired between P1 and P6 determined an increased neutralizing activity in MS-treated group. A possible explanation of this finding could be that, beside the Ab targeting the RDB domain, other Ab with a different specificity can contribute to the overall neutralizing activity. Indeed, anti-N-terminal domain (NTD) and anti-C terminal domain of S1 subunit were found to be nAbs in convalescent and vaccinated patients respectively, even if less prevalent than those targeting RBD (38–40). Along with neutralization, also S-specific CD4+ and CD8+ T cell response is increased in MS-treated group, suggesting that COVID-19 infection may increase both humoral and cellular immune response in these subjects. This phenomenon could relay first to the mechanism of actions of DMTs: the majority of patients showing this peculiar pattern were under the immunoreconstitution therapies alemtuzumab and cladribine, in which both T and B cells were depleted and then reconstitute toward a less inflammatory phenotype. Furthermore, the increase in IFNγ production can contribute to Ab affinity maturation, therefore augmenting Abs neutralizing efficacy (39).

Notably none of the pwMS under treatment with interferons experienced natural COVID-19 infection. Indeed, IFN-β administration has been related to a reduced viral load and a faster clearance of the mucosa, reducing the risk of severe disease (41–44).

The current study has several strengths. First, the usage of a pseudovirion-based neutralization assay to determine the real activity of elicited Ab. Secondly, the design of a prospective study allowed us to get a complete and detailed evaluation of humoral and T cell responses over time (up to 6 months after the third vaccine dose), in relation to specific DMTs taking into account the effects of likely confounding factors such as breakthrough infections.

A limitation of our work could be the size of each group resulting from the stratification of patients by therapy; however, as a monocentric longitudinal study, this cohort well represents the general MS population and the distribution of therapies used in clinical practice. Furthermore, we did not include analysis of B cell activation and phenotype; however, S-specific B-cell response was investigated in previous studies (45) showing reduced levels of B cell activity in pwMS under S1P modulators and anti-CD20 that is also influenced by post-vaccine anti-CD20 infusions.

Altogether, our observations combined with recent literature on the topic (2, 6, 8, 13–15, 26, 27, 32–35, 45) highlight the vaccine response data to current protocols applied in pwMS. The majority of pwMS under DMTs develop an efficient and long-term immune response comparable to HD. Collectively, fingolimod and ocrelizumab therapies show the lowest levels of protective immunity, underlying the necessity to carefully follow-up these subjects for the risk of a breakthrough SARS-CoV-2 infection and to have up-to-date vaccination coverage before starting these DMTs. Finally, we underline the necessity to rapidly generate a test combining Ab titers and neutralizing activity to determine which is the threshold required for protection to infection and/or severe COVID-19 disease.

## Data availability statement

The original contributions presented in the study are included in the article/**Supplementary Material**. Further inquiries can be directed to the corresponding author.

## Ethics statement

The studies involving human participants were reviewed and approved by the ethics committee of AOU San Luigi Gonzaga, Orbassano (TO), Italy (Ref. number #117-2021). The patients/participants provided their written informed consent to participate in this study.

## Author contributions

AM and RF: experimental design, interpreted the data; drafted the manuscript for intellectual content; IA and RR: major role in the acquisition and analysis of data; MM and MC: patient enrolment and follow-up: SR, MC and DL: designed and conceptualized the study; revised the manuscript for intellectual content. All authors contributed to the article and approved the submitted version.
