# Supplementary material for: Long-lasting neutralizing antibodies and T cell response after the third dose of mRNA anti-SARS-CoV-2 vaccine in multiple sclerosis
Source: Front Immunol. 2023 Jun 19;14:1205879. doi: 10.3389/fimmu.2023.1205879 (PMC10318111; doi:10.3389/fimmu.2023.1205879)
Supplement: Supplementary file 1 [file DataSheet_1.docx]

**Supplementary file**

**Long lasting neutralizing antibodies and T cell response after the third dose of mRNA anti-SARS-CoV-2 vaccine in multiple sclerosis**

Alessandro Maglione^a*^, Rachele Francese^b*^, Irene Arduino^b^, Rachele Rosso^a^, Manuela Matta^c^, Simona Rolla^a †^, David Lembo^b§^ and Marinella Clerico^a,c§^.

*^a^Department of Clinical and Biological Sciences, Laboratory of Neuroimmunology, University of Turin, Orbassano (TO), Italy;*

*^b^Department of Clinical and Biological Sciences, Laboratory of Molecular Virology and Antiviral Research, University of Turin, Orbassano (TO), Italy;*

*^c^ SSD Patologie Neurologiche Specialistiche, AOU San Luigi Gonzaga, Orbassano (TO), Italy*

*** AM and RF share co-first authorship

^§^ MC and DL share co-senior authorship

^†^Correspondence: Simona Rolla, email: [simona.rolla@unito.it](mailto:simona.rolla@unito.it), Department of Clinical and Biological Sciences, University of Turin, San Luigi Gonzaga Hospital, Regione Gonzole, 10 - 10043 Orbassano (TO) - Italy

**Supplementary materials and methods**

**VSV-G pseudotyped virus and plasmids**

G*ΔG-VSV-luc, a VSV-G pseudotyped virus bearing luciferase, and the plasmid encoding for the G protein (pCAGGS-G-Kan) were obtained from Kerafast (Boston, MA, USA, catalog number: EH1025-PM). G*ΔG-VSV-luc was amplified on BHK-21 cells transfected with pCAGGS-G-Kan plasmid, according to the manufacturer instructions1 and Nie et al [21].

The pcDNA3.1.S2 recombinant plasmid, which was constructed by inserting the codon-optimized S gene of SARS-CoV-2 (GenBank: MN908947) into pcDNA3.1 plasmid by using BamHI and Xba I restriction sites, was kindly provided by Prof. Weijin Huang, Miao Xu and Youchun Wang [21].

**SARS-CoV-2 pseudovirions production and titration**

SARS-CoV-2 pseudotyped viruses were produced and titrated according to Nie et al [21]. Briefly, 293T cells (5x105cells/ml in T-75 culture flask) were transfected with pcDNA3.1.S2 (30 µg) to overexpress the SARS-CoV-2 spike protein and infected with G*ΔG-VSV-luc (7x104 TCID50/ml) at the same time (Vf=15 ml). Two control flasks were produced in parallel: a mock control and a flask infected only with G*ΔG-VSV-luc. After an overnight incubation, the culture medium was aspirated and 2 gentle washes with PBS with 1% (vol/vol) FBS were performed. Fresh culture medium (DMEM supplemented with 10% FBS) was then added to the flasks and incubated for 24h at 37°C. The supernatants containing newly produced SARS-CoV-2 pseudovirions were collected at 24h, and at 48h after a second incubation. Supernatants were centrifuged at 1000g for 10 min at 4°C to remove cellular debris, filtered by using 0.45 µm filter and aliquoted in 2ml microtube for long term storage at -80°C. The production of G*ΔG-VSV-luc virions was performed under the same protocol by using the BHK21 cells and the pCAGGS-G-Kan plasmid. The titer of the new production was evaluated by means of the TCID50 (Median Tissue Culture Infectious Dose) assay. Briefly, 9 serial dilutions of SARS-CoV-2 pseudovirions (from 1:30 to 1:196830) were performed in sextuplicate in a 96-well plate. As uninfected cell control (CC), six wells were treated with culture medium alone. Subsequently, Huh7 cells were prepared at a final concentration of 1.7x105cell/ml and seeded 100 µl/well in the previously prepared 96-well plate. After a 24h incubation, the Britelite plus reporter gene assay system (PerkinElmer, Waltham, Massachusetts, USA, cat. no. 6066769) was added to cells in a 1:1 ratio with the culture medium (Vf = 200µl), for 2 min in the darkness at RT. 150µl of each well were then transferred to a corresponding 96-well chemiluminescence detection plate and the RLU (relative light units) were read in the Infinite F200 luminescence reader (TECAN, Männedorf, Switzerland). Positive wells were identified as wells presenting 10 times the average RLU of the CC wells and the TCID50 value was calculated based on the positive and negative rate in each dilution, according to the Reed-Muench method.

**Pseudovirions characterization**

SARS-CoV-2 pseudovirions were analyzed by means of Western Blot analysis to verify the presence of the SARS-CoV-2 spike protein on the VSV envelope [30]. SARS-CoV-2 pseudovirions (8 ml) and G*ΔG-VSV-luc virions (8 ml) were pelleted on 25% (w/v) sucrose cushion via ultracentrifugation at 100 000g for 2h at 4°C, and then viral pellet was resuspended in 100 µl of PBS 1x. A small aliquot of concentrated pseudovirions (15 µl) was subsequently resuspended and lysed in SDS loading buffer and incubated in a heating block at 95°C for 5 min. For immunoblotting, proteins were separated by sodium dodecyl sulfate–8.5% polyacrylamide gel electrophoresis (SDS-8.5% PAGE) and transferred to polyvinylidene difluoride (PVDF) membrane. Membranes were then incubated overnight with blocking buffer consisting of PBS, 0.1% Tween 20, and milk powder 10% and immunostained with the following Abs: anti-SARS-CoV/SARS-CoV-2 (COVID-19) spike antibody [1A9] (Genetex, Irvine, California, USA) and anti-VSV-M [23H12] antibody (Kerafast, catalog number: EB0011). Immunocomplexes were detected using the secondary antibody peroxidase-conjugated AffiniPure F(ab’)2 Fragment goat anti-mouse IgG (H + L) (Jackson ImmunoResearch Laboratories Inc., West Grove, PA, USA).

**References**

[21] Nie J, Li Q, Wu J, Zhao C, Hao H, Liu H, et al. Quantification of SARS-CoV-2 neutralizing antibody by a pseudotyped virus-based assay. Nat Protoc 2020;15:3699–715. https://doi.org/10.1038/s41596-020-0394-5.

[30] Whitt MA. Generation of VSV pseudotypes using recombinant ΔG-VSV for studies on virus entry, identification of entry inhibitors, and immune responses to vaccines. J Virol Methods 2010;169:365–74. https://doi.org/10.1016/j.jviromet.2010.08.006


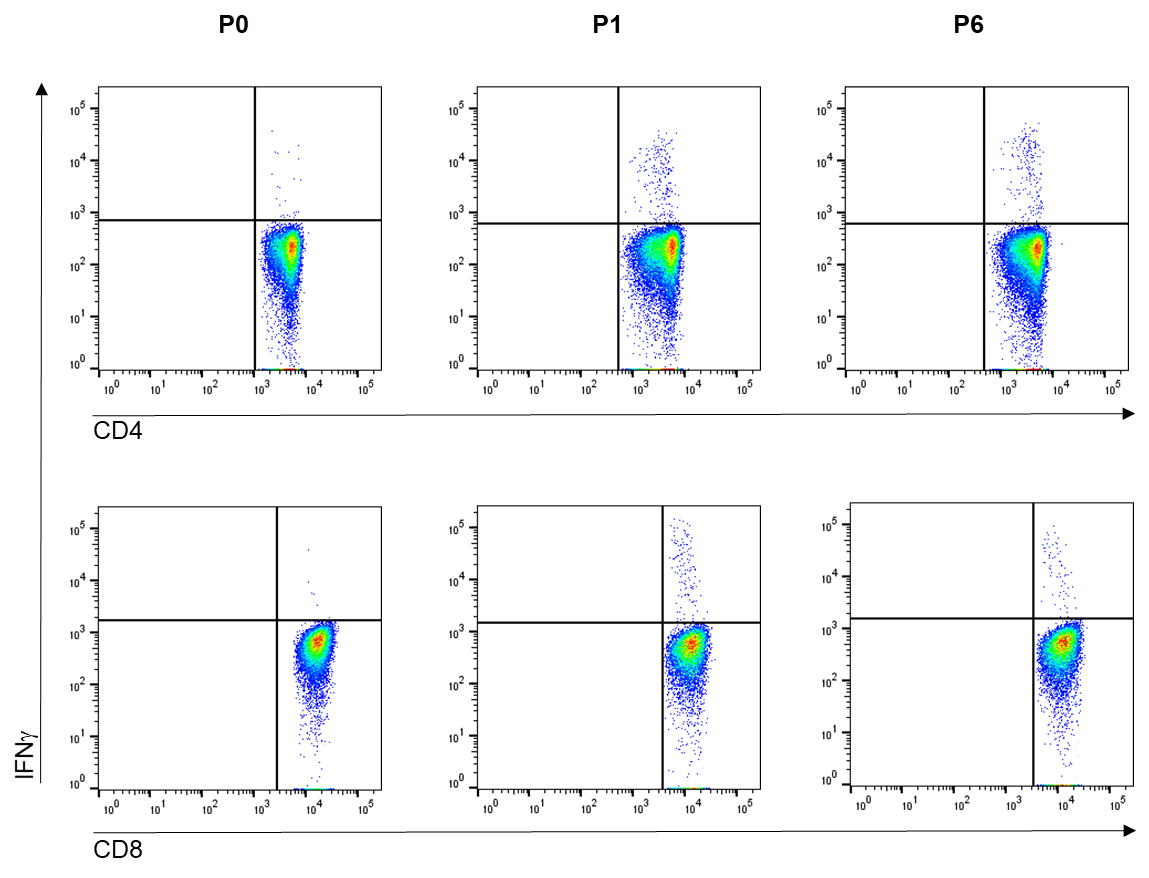


**Supplementary figure 1:** Representative dot plots of Spike specific IFNγ-CD4 (upper panels) and CD8 (lower panels) T cells before (P0), 1 (P1) and 6 (P6) months after the three doses of anti-SARS-CoV-2 vaccine.
